# Creation of a novel simple heat mapping method for curriculum mapping, using pathology teaching as the exemplar

**DOI:** 10.1186/s12909-021-02808-3

**Published:** 2021-07-08

**Authors:** Ryan Clark, Sarah Bell, Jennifer Roccisana, Karin A. Oien, Sharon F. Sneddon

**Affiliations:** 1 School of Medicine Dentistry and Nursing, College of Medical Veterinary and Life Sciences, University of Glasgow, Wolfson Medical School Building, University Avenue, Glasgow, G12 8QQ UK; 2 Pathology Department, Queen Elizabeth University Hospital, Glasgow, UK; 3 Institute of Cancer Sciences, College of Medical Veterinary and Life Sciences, University of Glasgow, Glasgow, UK

**Keywords:** Curriculum mapping, Pathology, Curriculum, Heat maps, Undergraduate medical education

## Abstract

**Background:**

The undergraduate five-year MBChB programme at the University of Glasgow has a high volume of pathology teaching integrated into the course. The ability to better understand what pathology is taught and when, so as to build a picture of the types and depth of pathology topics covered across the programme stages is crucial, especially in a spiral curriculum. A novel method of curriculum mapping, known as curriculum heat mapping, was developed as a way to visualise where and when topics are taught, in an easier to understand format.

**Methods:**

This method involved comparing the Glasgow curriculum to a pre-determined standard of what should be taught. In this case, The Royal College of Pathologists’ ‘Pathology Undergraduate Curriculum’ was used as a comparison of what a graduating doctor should know about pathology.

**Results:**

Following the developed template, heat maps showcasing the range of pathology topics covered, and where they are covered, were developed for local use.

These heat maps provided a clear visual representation of where and when topics are taught, and how they cluster.

**Conclusions:**

Heat mapping is a novel low-cost, high-input method of curriculum mapping. It requires a person to input the data which can take a long time for large curricula. There are no other upfront financial costs. It can be used in any area with a curriculum and an external or internal comparator. Examples of gold standard external comparators include validated national or international curricula.

Heat mapping can help integrated, spiral curriculum programmes to identify where core topics are taught throughout their course. The heat maps themselves successfully demonstrate the required information and are easy to interpret. The process of mapping, as well as the final heat map, can yield important information. This includes information about trends within the curriculum, areas for potential improvement in sessional design and a clearer understanding of the depth to which each topic is covered in each lecture.

Overall, it is a viable novel method, which has been successful locally and is easily transferable to other areas such as pharmacology.

**Supplementary Information:**

The online version contains supplementary material available at 10.1186/s12909-021-02808-3.

## Background

**Fig. 1:**
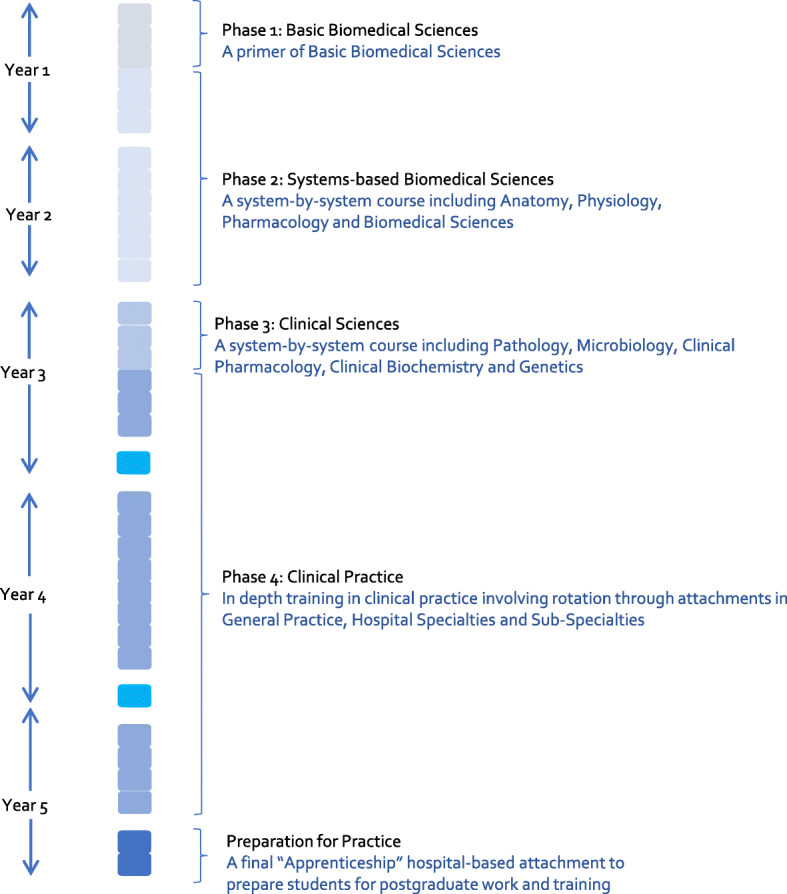
The structure of the University of Glasgow’s MBChB degree. The structure has been broken down by phase and showing rough timings for each phase. For example, phase one lasts for half of the first academic year and thus covers half of the first-year box

The MBChB at the University of Glasgow (henceforth Glasgow) is a 5-year undergraduate programme of study [1] in the United Kingdom. The programme of study, which results in the award of the MBChB degree, is composed of a spiral curriculum comprising of four phases followed by a Preparation for Practice block, as shown in Fig. 1, [1]. The curriculum can be defined as the content used to make up the programme of study, which follows a syllabus of intended learning outcomes (ILOs). The spiral nature of the curriculum introduces and revisits concepts throughout the course, in greater depth and complexity [1, 2].

Phase 3 is known colloquially as the ‘pathology phase’, as it is traditionally considered to be where pathological concepts are taught in detail by practicing pathologists and clinicians [1]. The discipline of pathology at Glasgow covers the underpinning mechanisms of why disease occurs. The spiral curriculum approach aims to enable learning incrementally from the early years through to Phase 3 and beyond. Thus, many concepts underpinning pathology, for example wound healing, are first taught earlier, in Phases 1 and 2. This strong integration of pathology within biomedical science modules by its nature brings challenges in identifying pathology-related teaching across the programme for more discipline-level curriculum development and review. It would therefore be useful to have a simple method to gain a clear overview of what pathology-related topics are taught, to what depth and at what stages.

Curriculum mapping (CM) is one possible method. A full review of CM can be found in AMEE guide 21 [3]. Briefly, CM is a useful way of representing the different elements of curricula to show links between them [3, 4]. Curriculum maps can be used to plan changes or highlight gaps and overlaps in curricula, as well as being useful to students to help identify what is being taught and where [3, 4]. While there are many methods for carrying out CM, one of the most commonly used is curriculum mind maps (CMM) [3, 4]. CMMs are extensive, branching figures which show the interdependence of different parts of courses, such as teaching methods and assessment. While these extensive maps can be useful, a large amount of time is required to plan, visualise, implement and analyse a map [3, 4]. Therefore, potential other methods of visualising the pathology curriculum were considered.

Heat mapping was chosen as a way to show complex data effectively. While heat mapping is not a commonly used curriculum mapping method, it does appear occasionally in the literature [5, 6]. In a previous study by one of the authors, simple heat mapping was used to map whole courses to UK-specific [5]. As a consequence of this, the concept of grids of colour representing topics covered, and where they were covered, seemed appealing as a way of curriculum mapping. Therefore, this study considered how to develop simple heat mapping, in the context of pathology teaching.

This study thus describes a novel low-cost, high-input CM method, with pathology as an exemplar. The aims were to design a novel method of CM using heat mapping and then to develop this method by applying it to the Glasgow undergraduate medical curriculum, using pathology-related teaching as an exemplar.

## Methods

Heat mapping was used to provide a clear visualisation of where and what pathology is covered across Phases 1, 2 and 3 of the Glasgow MBChB curriculum (see Fig. 1).

### Resources

Heat mapping requires two curricula. First, there is the curriculum under study, which here is the Glasgow curriculum. Second, there is a pre-determined standard for comparison. The ‘Pathology Undergraduate Curriculum’ (henceforth RCPath curriculum) of the UK’s Royal College of Pathologists (RCPath), in its first edition, published in 2014 [7] was used. Microsoft Excel was used as the software to create the heat maps. A custom database table was built with the Glasgow curriculum as rows in an Excel worksheet and the RCPath curriculum domains as columns in Excel. This Excel worksheet/database enables data capture, comparison, analysis and visualisation.

### Approach

Curriculum mapping requires decisions to be made early on about the level of learning outcome used for comparison and therefore for initial data capture. This requires deciding on the curriculum to be examined and the curriculum to compare it against. Once this has been decided, identifying the level of learning outcome which provides the necessary data is essential to constructing the Excel worksheet. A stepwise guide to completing a heat mapping exercise is shown in Additional File 1.

#### RCPath curriculum as pre-determined standard; and level used for comparison

**Fig. 2:**
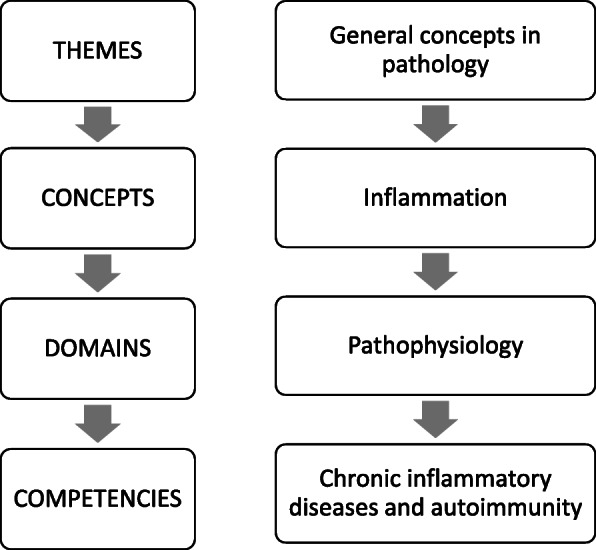
Flowchart showing how RCPath 2014 Pathology Undergraduate Curriculum may be sub-divided to enable curriculum mapping. The RCPath Curriculum describes broad themes (such as general concepts in pathology). These themes are then sub-divided into different concepts (such as inflammation, or cardiovascular system). Each concept is then sub-divided by the different sub-specialist domains in pathology (pathophysiology, microbiology, haematology and biochemistry). For each of these concept/domain combinations, finally, competencies (similar to intended learning outcomes) are listed. This creates a system where one or more competencies are listed for the concepts, divided by domains, for example, the pathophysiology of inflammation. For our research, we used the level of the domains within each concept to compare to our Glasgow curriculum: this meant that we compared teaching sessions to RCPath concept/domains; and we defined what was actually in each concept/domain by its competencies listed in the RCPath Curriculum

For the pre-determined standard, the RCPath curriculum was used. As its introduction states: “*The curriculum has been designed to define a core level of knowledge, skills and behaviours for foundation doctors, i.e. a minimum level of training, relating broadly to all aspects of Pathology, at medical school*” [7]. The curriculum lists competencies which are categorised in broadly in two ways and specifically by area of pathology (Fig. 2). First, pathology learning is divided, as has been traditional, into themes of: (1) knowledge relating to the cell biology, pathology and clinical features of biological processes, known as “general” or “basic” pathology; (2) knowledge relating to systems of the body, known as “systemic” pathology; and (3) general competencies. These are further sub-divided, for example (1) “general” pathology includes: inflammation; infection; neoplasia; etc. Second, different pathology disciplines are described in four so-called “domains”: pathophysiology (pathology); microbiology; haematology; and biochemistry. For each theme/domain sub-category, the competencies include knowledge, skills and/or behaviours. These competencies equate to Intended Learning Outcomes (ILOs).

The RCPath curriculum provides a concise yet detailed examination of the areas within pathology which should be covered and what topics constitute these areas. Data was examined by grouping the competencies by domain, for example, inflammation pathophysiology. This was chosen to provide sufficient detail for analysis, without making the worksheet too complicated, and it was felt to be achievable within the timescale available. This level provided 54 items for comparison.

#### Glasgow curriculum under study; and level used for comparison

For the Glasgow curriculum, Phases 1–3 were the focus as this was where the majority of pathology was taught (Fig. 1). Sessions were removed which did not provide curriculum-specific content (administrative/pastoral sessions) or which were not core classes (elective modules and optional research years).

The Glasgow curriculum is structured into phases, blocks within a phase, weeks within a block and then individual sessions within a week (Fig. 1). Thus, the course was examined at the individual session level. Intended learning outcomes (ILOs) for each individual session as a surrogate for the session content were used. ILOs are a brief list of what a student should know/be able to do after the session and so should summarise the content of the session [8]. ILOs were acquired from the online learning environment (Moodle) 2017-18 academic year pages where ILOs are provided for each week. 502 sessions were identified for consideration. In two sessions, lecture slides were considered as the ILOs did not provide sufficiently clear evidence of coverage. This decision was taken on the basis that the ILOs were high-level and thus would apply to most RCPath competencies: it was decided that checking if the session content fully matched the high-level ILOs was necessary to justify mapping it to the competencies.

#### Excel spreadsheet: initial customisation and data capture

These two curricula were used to create a custom worksheet in Microsoft Excel with the RCPath domains as the Excel columns and the Glasgow individual sessions as the Excel rows. To reduce the size of the worksheet, the Glasgow curriculum sessions was split into the different phases: all the sessions of each phase had their own tab within the spreadsheet. This was done to help with data entry. All tabs had all 54 columns from the RCPath curriculum, as 54 columns was deemed workable whereas 502 individual sessions in one sheet was found to be unwieldy.

Once the tabs were set-up for data entry, the two curricula were compared using a six-stage process:

1. Identify session title and ILOs.
2. Consider what area of biomedical sciences are included.
3. Match these to the areas of pathology relevant to this subject.
4. Consider if these areas are within the ILOs listed.
5. Check every domain with the ILOs, as an addition to 4.
6. Match these to the value judgement: ‘*is this pathological content, is this nested pathological content or is this not pathology*?’

The aim was to identify, for each Glasgow session, whether any of the RCPath domains were addressed and if so which. This process was chosen to ensure all pathology content was captured. If pathology is taught in a clinical lecture rather than by a pathologist, this could be missed simply by comparing the intent of the lecture with the domains. Using this six-step process allowed for the concern about missing pathology to be directly addressed in the methodology of the study, as this potential is directly accounted for.

Following this process, if a Glasgow session met the RCPath domain, a 1 was placed in the cell in Excel. If it did not, it was left blank to represent a 0. This was done for all 502 sessions. This provided a complete-overall-spreadsheet dataset of comparisons between teaching sessions and domains.

#### Excel spreadsheet: curriculum data analysis and heat map visualisation

Once the complete-overall-spreadsheet was assembled, it was converted to a more user-friendly data set to allow a user to see how individual sessions built a complete curriculum and how well this matched the RCPath curriculum:

To do this, individual session numbers were converted into their composite weeks. This summing into weeks allowed a striking visual – but not overwhelming – presentation of the data. The Excel SUM function was used to add all the sessions in a week into one single row. A new row after all the sessions in a week (called Week # summation) and added together the ‘1’s for each domain in that week. This provided a row for each week, with 54 numbers, which corresponded to the number of sessions which met the RCPath domains.

These summations were then taken to a new tab in the spreadsheet to give a tab which covered each domain per week rather than per session. Once they were in the new spreadsheet, conditional formatting was used to provide a scale of hues of blues. The darker blues indicated that week had more sessions where the ILOs met the RCPath competencies within the RCPath. Finally, at the bottom of that spreadsheet, all the values for each week were added together to give a single line indicator of the number of times each domain was met within the Glasgow curriculum data as a whole. This was then separately conditionally formatted to show the relative comparisons between each domain across the Glasgow curriculum.

#### Validation of results

A three-stage approach to validation was undertaken. Intra-educator and intra-observer variability were assessed followed by examination of how well the ILOs matched the teaching content on a sample of sessions.

## Results

### Outputs from this research

The outputs from this research were twofold: the curriculum heat maps themselves and the lessons learned from the heat mapping process.

### Notes about analysis of the heat maps

Prior to analysis, 26 teaching sessions were removed from the spreadsheet due to lack of relevance. Three weeks of the course were removed as they were not considered teaching weeks in the 2017-18 curriculum. All 502 teaching sessions were then compared to the 54 RCPath domains, providing 27,108 data points for analysis. These data were used to create heatmaps to enable curricula to be visualised and reviewed.

When using conditional formatting for analysis of heat maps, the maximum possible hue available is key to interpreting the maps. The 502 sessions were broken by phase and week to give an indication of the maximum amount of pathology, and therefore the maximum hue available, for each domain per week. For example, Phase 1-week 6 has 6 sessions whereas Phase 3-week 12 has 15 sessions. The latter can therefore have a darker maximum hue than the former. As a result, in performing analysis of the results, the potential maximum hue of the different weeks can vary. Any analysis must be cognisant of this fact: the exact potential depth of coverage per week is different and thus detailed analysis of depth of coverage cannot be extrapolated from the heat mapping. However, it can give a simple understanding of rough depth (the darker it is, the more it is covered) and reasonable indication of where topics are clustered. These areas can be examined further to determine depth of coverage and method of coverage by triangulating the map with ILOs, slides or recordings of the lecture.

### Heat maps

**Fig. 3:**
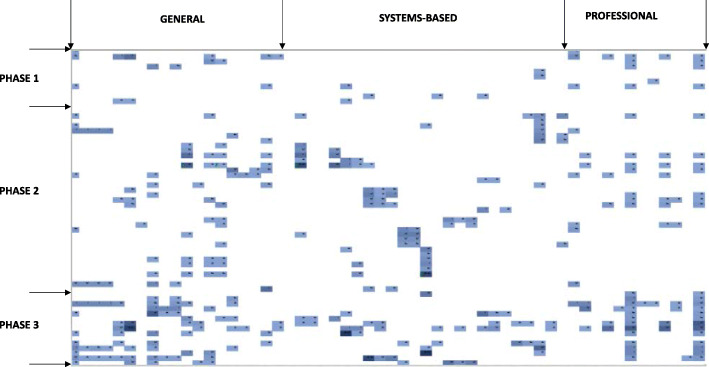
Week-by-week curriculum heat map. This heat map showing where and when topics were taught, with darker blue hues indication greater relative number of sessions covering the topic. This heat map breaks the phases down into each week contained within them. It breaks the comparison curriculum into each field within the Undergraduate Pathology Curriculum. These are grouped, for ease of understanding into their constituent domains

**Fig. 4:**
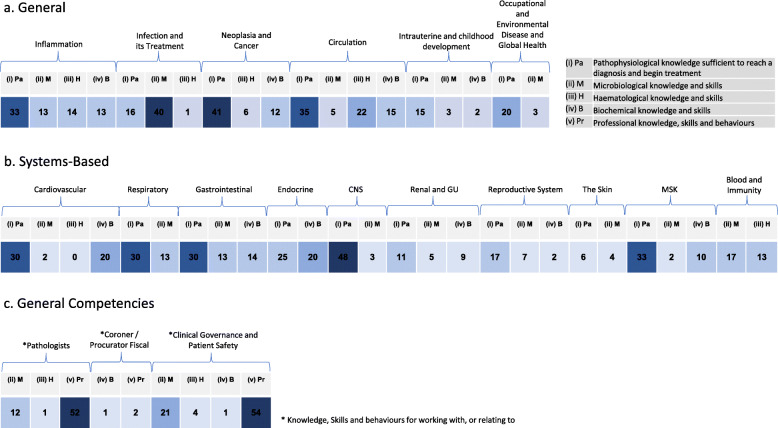
Overview curriculum heat maps. Overview heat maps showing the absolute number of sessions covering a field across the first three phases of the curriculum at the University of Glasgow medical school. This allows assessment of the overall coverage of a topic including identifying gaps in the curriculum and examining how much resource goes into a specific field. **a** Depicts the General theme, **b** Systems Based and **c** Professional Domains according to the RCPath themes

The global map in Fig. 3 shows the overall heat mapping by phase of the Glasgow curriculum and by theme of the RCPath curriculum. This global summary was condensed into a single line (Fig. 4) to allow a simple indication of what was, and was not, covered in the curriculum and how many times a domain was addressed across all three phases. These two figures were used as the basis for initial analysis of the curriculum. Further subdivision of the heat maps is possible.

The spread of pathology across the phases and across the themes, in Fig. 3, highlights the diversity of topics covered over time within the Glasgow curriculum. Phase 3 has the majority of the pathology teaching and Phase 2 clusters pathology specifically from system-based pathology. Phase 1 has a small volume of pathology. There are several other clusters, notably general pathology in Phase 3 and professional attributes in Phase 3.

Figure 3 shows the full heat map. Most of the “fields” are covered extensively in Phase 3 with some back spread into the earlier phases. Some of the fields are covered earlier and less so in Phase 3. This is mostly due to the breakdown of the content: pathophysiology of infection, for example, covers the structure of bacteria and viruses, as well as the pathogenesis and sequelae of infections like HIV which would vary in coverage across the phases. This mix of basic sciences and pathology means that coverage will be across the curriculum, but the exact ILOs covered within each session for a particular field may be different. Thus, the mapping tool allows identification of how a strand of learning builds across the curriculum, in an easily identifiable format. This allows topic leads to trace how a student’s knowledge builds across the years and examine how to optimise this spiral stranding.

Figure 4 provides a clear picture of the overall coverage of each field. This figure was created by merging all of the data in Fig. 3. In the Glasgow curriculum, it highlights that every topic is covered with the exception of ‘superior vena cava obstruction’ in Phases 1–3. This was later confirmed to be covered in Phase 4, and therefore not captured in this exercise.

### Map creation itself yields helpful information

The process of creating and producing the heat maps was useful in itself. It allowed several trends, which were not necessarily highlighted in the final heat map, to be identified. These are equally important for curriculum development. Firstly, it was noted there was a progression in thematic content throughout the phases. Lecture ILOs and titles evidenced the concept of a spiral curriculum: for example, a session in Phase 1 on respiratory physiology is developed further in a gas exchange and transport lecture in Phase 2 and then discussed in a clinical content in Phase 3.

Second, sometimes the progression created partial or complete overlap of content. One such example is a similarly titled lecture which was delivered in both Year 2 Phase 2 and Phase 3. Both lectures are given by the same lecturer and use mostly the same slides. However, the ILOs for the lectures differ. This would be considered in this report to be complete overlap. Lectures within the same phase cover this topic from different angles and across phases some lecture content is duplicated.

## Discussion

### Lessons from the pathology curriculum

Harden’s definition of a curriculum, expanded upon by Al-Ehd et al., explains that a curriculum should be many educational strategies which are easily communicable to staff and students [3, 4, 6]. Glasgow’s curriculum is spiral in nature, using blocks to group content to make the concepts relevant to each system easy to understand and find. In Phase 1, there are small amounts of pathology scattered throughout the biomedical sciences. Phase 2 shows an increased amount of pathology, with clusters of heat (the blue hues) around the blocks relevant to the domain. This builds two pictures for the curriculum: firstly, there is a progression of volume of pathology across the phases (in keeping with the spiral curriculum) and secondly that pathological concepts are clustered around the weeks that are relevant to them, as one would expect. Phase 3 further strengthens the evidence for these two lessons. Clearly, the design philosophy for the curriculum is being adhered to. Whether this leads to a set of behaviours which are hoped for (level 3 evaluation on Kirkpatrick’s scale [9]) is something which could be determined in future research.

The process of creating the maps provided several points for consideration which were not evident from the final map. The indication that both the mapping process itself and the final maps are useful strengthens the effectiveness of this methodology for providing applicable, pragmatic lessons for curriculum development. Being able to consider both progression of topics and sessional overlap in a more detailed fashion than the generalised maps provides a way to discuss the purpose of the sessions in the course and identify areas for improvement. This can then drive curriculum development independent of the trends in the maps. For example, in the Glasgow curriculum, pathology is covered in good detail and thus multiple similar sessions may not be needed to adequately cover the ILOs. Instead, this session could be used to supplement other areas of the curriculum. While session by session consideration may be a small change – an hour or so every so often – with curricula under time pressures, these small volumes of time can be extremely useful.

Interpreting the final heat maps to influence local curriculum development must be done while considering the nature of the comparisons being made. The data is a comparison to itself rather than a pre-defined standard. This means the intensity of the hue is decided based on the numbers available rather than on a theoretical maximum. It would be possible, but perhaps not useful, to set a particular number as the standard for each column (domain) and compare the numbers in each week to this. The clusters of heat would be less useful here as the data would need to be considered column by column rather than as a collective as there would be no collective comparison. However, when the maps are considered with these caveats in mind, the final maps themselves provide a useful indication of where and when topics are taught and how they cluster.

### The benefits and limitations of using heat mapping for curriculum mapping

The benefits of heat mapping are multiple. Firstly, mapping the pathology curriculum using heat maps makes it easier interpret for staff and students and is transferrable to other specialties. In particular, it is clear that this tool can (1) highlight stranding of key topics within a curriculum and (2) provide a way for leads to easily identify sessions which cover their topic so that they may review those together to see how a student’s knowledge is built as the course progresses. The results prior gave a good indication of the level of detail that can be achieved through using heat mapping for examining coverage of pathology within medical curricula. This is very easily adaptable for any curriculum, at undergraduate or postgraduate level. For example The British Pharmacological Society have produced an undergraduate curriculum for pharmacology students which could be modified and used in a similar way [10]. It similarly has three domains, but the sub-structure is different which would require slight optimisation of the database cells in order to be utilised. However, the same principles would apply, and the output could be used in similar ways or even compared to other disciplines such as pathology to understand the overlap and differences between the courses.

Secondly, the heat map provided a low-cost, high-input method of mapping a curriculum. One example of a commercial curriculum mapping tool has a £12,000 set-up fee and a £6000 yearly licence [11]. It also has an additional £12.50 per student per year fee if students want to use it for learning [11]. While this tool has a variety of useful features, part of its function can be completed with heat mapping. In particular, especially in developing areas which may not have access to the funds needed for this software, Microsoft Excel spreadsheets for heat mapping is a very viable alternative. Even for medical schools in developed countries, the use of the heat mapping process and final maps is beneficial due to its ease of interpretation. Locally, busy clinicians have found a striking single map to help them conceptualise where and when teaching occurs, and this has helped start conversations about quality improvement. While this method trades financial costs for the opportunity cost of what the person could be doing in the mapping time instead, the wealth of benefits makes the tool useful. It could be used alongside software such as that described above or instead of it and thus provides a viable alternative to mapping which is clear, easy to visualise and useful for engaging busy clinicians with little time to learn how to operate new software.

Heat mapping requires a large upfront time cost to complete the mapping process. In the Glasgow curriculum, ILOs are split into different documents for different areas of the course. Thus, all the ILOs, construction of the maps, completion of the data entry and analysis resulted in a data set that amounted to 27,108 data points. This was completed over a three-week period; however, depending on decisions around sessions matching to the comparator curriculum, it may take longer. Thus, while the benefits are worthwhile, finding an academic or student with the time to undertake data entry and analysis such as this may be different.

Furthermore, an inherent limitation of this work is the subjective nature of the comparisons being made. This can be considered on two levels: the session-by-session subjective comparisons to the RCPath domains and the perfect scenario ILOs present as to what is actually taught (discussed further below). Validation of the results was carried out to ensure the method matched expectations, but due to the short timeframe of the student project, there were limitations in the scale of the validation we were able to carry out. This study benefited in that the person making these decisions was a student who had recently completed these classes and thus knew much more clearly what each ILO meant with regards to the session content. Potentially, this makes it an attractive method for student involvement in medical educational research but, if a single academic has to complete this without intimate knowledge of the course for consideration (i.e. the Glasgow curriculum in this case), this may cause issues in the usability of the data.

### Assessing the methodology underpinning heat mapping

While the benefits and limitations of the process and the final maps helps us understand the usefulness of this method, considerations of the components of the methods is equally important to ensuring the method is sound. When designing this study, different surrogates for the session content were considered: ILOs, slides and general written lecture summaries are some examples. A balance was required between depth of understanding of the session content and usability of the surrogate within the timeframe for project completion. ILOs were considered to be a good balance. However, there needs to be consideration as to the appropriateness of ILOs as a surrogate of session content. Harden asserts that ILOs, when written clearly, allow content providers a way to know the content of their session [8]. Therefore, provided, at Glasgow, these ILOs were written clearly, they would be an appropriate to use ILOs as a surrogate measure of session content for this project. For the purposes of this project, a detailed analysis of ILOs was not carried out. However, this is an area for future consideration at a local curriculum development level.

This process of using ILOs works in so far as it is a best-case scenario. There is no guarantee that lecturers will follow the ILOs strictly or that the ILOs will be a good reference of the depth of content within the session. For example, an ILO to ‘recognise’ what a condition is will be sufficiently less detailed than an ‘explain’ ILO on rheumatoid arthritis. This project therefore gives equal weight to the different ILOs and this could mean that some of the coverage is overestimated. However, without being present in each session, it is difficult to gain a better appreciation of the contents covered. Doing that would also be too time consuming compared to using the ILOs for little additional benefit.

The RCPath document was used due to its standardised layout, the fact it was agreed across the UK and because it was from the body which sets professional and training standards for pathologists within the UK [7]. Furthermore, 54 outcomes provided sufficient detail of the spread of coverage of pathological concepts [7]. The concise nature of the document was useful for the most part in creating something which was quickly understandable and useable. On the other hand, the brevity meant that some phrases or statements were rather wide in scope so that clarification was helpful. For example, ‘primary muscle diseases’ was listed as a competency: to understand what teaching topics that might include, a standard undergraduate pathology textbook was consulted. Despite these minor limitations, the strengths of this document ensured that it was the best comparison for this project. If this was to be extended to other areas, a standardised national set of expectations like this could be a gold-standard.

### Heat mapping depth considerations

Finally, the question of depth of coverage of topics within the Glasgow course was hotly debated in the design of this method. Heat mapping can only provide a limited view of depth: it can show the number of sessions in a week which cover a topic but not the depth of coverage within each of those sessions. Originally, there was discussion around using a tiered system for grading the sessions: 0 for no coverage, 1 for some coverage, 2 for moderate and 3 for detailed coverage. However, in the timeframe available, this level of analysis was not possible. There is some evidence that assigning this level of coverage is little different to using a binary scale [12]. However, as depth is a matter of interest for educators, further research is ongoing into how to incorporate depth into the template as an additional later series of steps.

## Conclusions

Curriculum mapping has the potential to inform students and staff in the way they learn and teach. This study has helped understand the where and when of pathology teaching across the Glasgow undergraduate curriculum and how this aligns with the RCPath curriculum. It has highlighted areas for improvement in the curriculum as well as gaps in teaching. It is envisaged that it will be easily transferable to other areas of the Glasgow curriculum such as pharmacology and will also be applicable to the postgraduate setting. Future research is required to examine incorporating depth of teaching, implementation of heat maps into development of curricula and how to use heat mapping in specific other subjects such as pharmacology.

## Supplementary Information

**Additional file 1.** A complete template for carrying out a own heat mapping exercise. This template identifies each step in completing a heat mapping exercise and can be followed for developing heat maps.

## Data Availability

The data that support the findings of this study – namely the intended learning outcomes relevant to each session - are available from the University of Glasgow but restrictions apply to the availability of these data. Data are however available from the authors upon reasonable request and with the permission of the University of Glasgow.

## Abbreviations

CM: Curriculum mapping
CMM: Curriculum mind maps
ILOs: Intended learning outcomes
RCPath: UK’s Royal College of Pathologists
RCPath curriculum: The ‘Pathology Undergraduate Curriculum’ of the UK’s Royal College of Pathologists
