# Supplementary material for: Creation of a novel simple heat mapping method for curriculum mapping, using pathology teaching as the exemplar
Source: BMC Med Educ. 2021 Jul 8;21:371. doi: 10.1186/s12909-021-02808-3 (PMC8265068; doi:10.1186/s12909-021-02808-3)
Supplement: Supplementary file 1 — Additional file 1. A complete template for carrying out a own heat mapping exercise. This template identifies each step in completing a heat mapping exercise and can be followed for developing heat maps. [file 12909_2021_2808_MOESM1_ESM.pptx]

## Slide 1
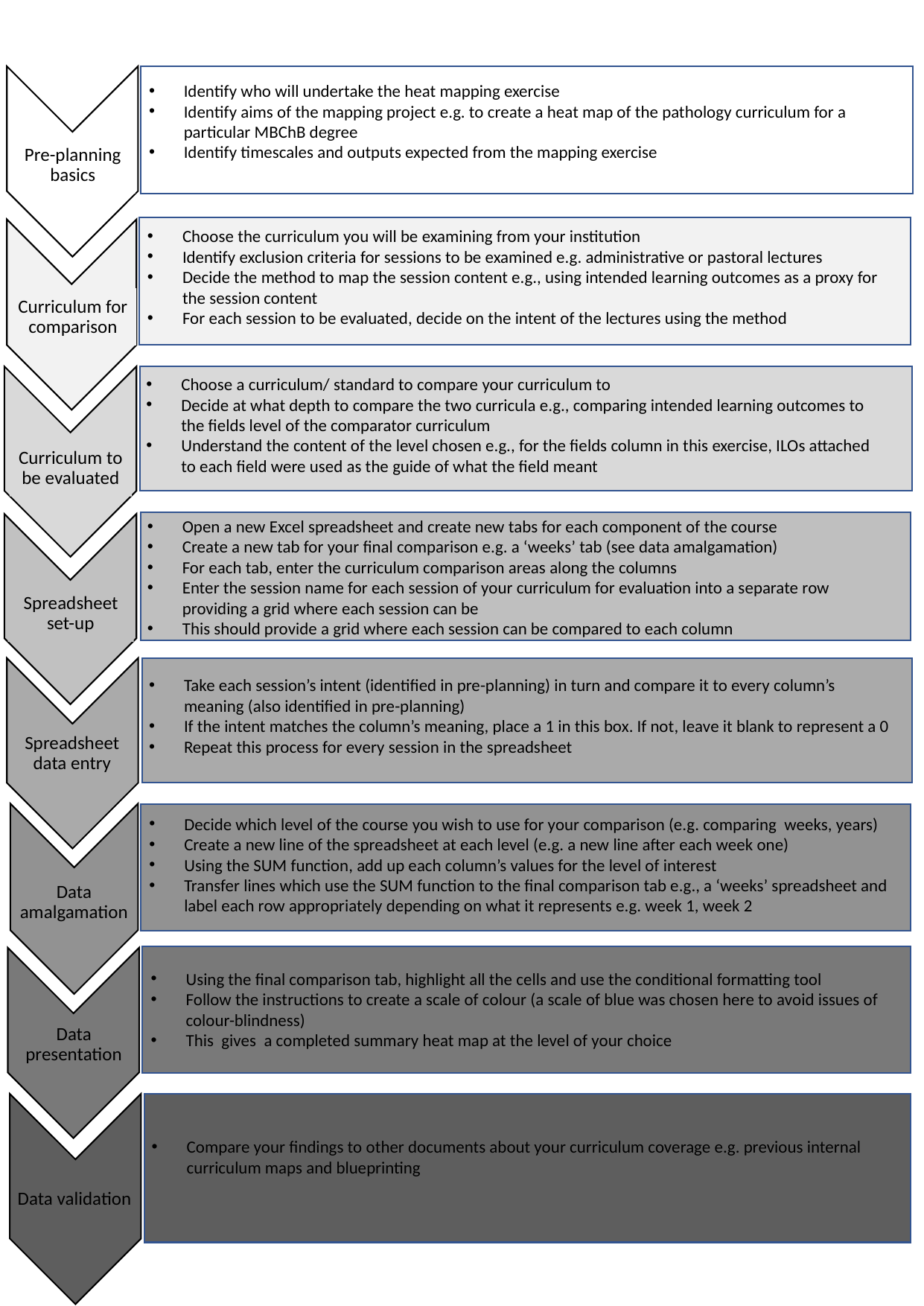

Pre-planning basics
Identify who will undertake the heat mapping exercise
Identify aims of the mapping project e.g. to create a heat map of the pathology curriculum for a particular MBChB degree
Identify timescales and outputs expected from the mapping exercise
Curriculum for comparison
Choose the curriculum you will be examining from your institution
Identify exclusion criteria for sessions to be examined e.g. administrative or pastoral lectures
Decide the method to map the session content e.g., using intended learning outcomes as a proxy for the session content
For each session to be evaluated, decide on the intent of the lectures using the method
Curriculum to be evaluated
Choose a curriculum/ standard to compare your curriculum to
Decide at what depth to compare the two curricula e.g., comparing intended learning outcomes to the fields level of the comparator curriculum
Understand the content of the level chosen e.g., for the fields column in this exercise, ILOs attached to each field were used as the guide of what the field meant
Open a new Excel spreadsheet and create new tabs for each component of the course
Create a new tab for your final comparison e.g. a ‘weeks’ tab (see data amalgamation)
For each tab, enter the curriculum comparison areas along the columns
Enter the session name for each session of your curriculum for evaluation into a separate row providing a grid where each session can be
This should provide a grid where each session can be compared to each column
Spreadsheet set-up
Spreadsheet data entry
Take each session’s intent (identified in pre-planning) in turn and compare it to every column’s meaning (also identified in pre-planning)
If the intent matches the column’s meaning, place a 1 in this box. If not, leave it blank to represent a 0
Repeat this process for every session in the spreadsheet
Data amalgamation
Decide which level of the course you wish to use for your comparison (e.g. comparing weeks, years)
Create a new line of the spreadsheet at each level (e.g. a new line after each week one)
Using the SUM function, add up each column’s values for the level of interest
Transfer lines which use the SUM function to the final comparison tab e.g., a ‘weeks’ spreadsheet and label each row appropriately depending on what it represents e.g. week 1, week 2
Data presentation
Using the final comparison tab, highlight all the cells and use the conditional formatting tool
Follow the instructions to create a scale of colour (a scale of blue was chosen here to avoid issues of colour-blindness)
This gives a completed summary heat map at the level of your choice
Data validation
Compare your findings to other documents about your curriculum coverage e.g. previous internal curriculum maps and blueprinting
